# Effect of Continuous Glucose Monitoring Following Hospital Discharge of Patients With Type 2 Diabetes

**DOI:** 10.1210/jendso/bvaf169

**Published:** 2025-11-05

**Authors:** Cecilia Wallace, Melanie Natasha Rayan, Sara Folk, Cara Harris, Eileen Faulds, Thaina Gatti, Philicia Duncan, Elizabeth Buschur, Kathleen Wyne, Trevor Sobol, Jianing Ma, Kathleen M Dungan

**Affiliations:** The Ohio State University College of Medicine, Columbus, OH 43210, USA; Division of Endocrinology, Diabetes and Metabolism, College of Medicine, The Ohio State University Wexner Medical Center, Columbus, OH 43210, USA; The Ohio State University College of Medicine, Columbus, OH 43210, USA; Division of Endocrinology, Diabetes and Metabolism, College of Medicine, The Ohio State University Wexner Medical Center, Columbus, OH 43210, USA; Division of Endocrinology, Diabetes and Metabolism, College of Medicine, The Ohio State University Wexner Medical Center, Columbus, OH 43210, USA; Division of Endocrinology, Diabetes and Metabolism, College of Medicine, The Ohio State University Wexner Medical Center, Columbus, OH 43210, USA; Division of Hospital Medicine, College of Medicine, The Ohio State University Wexner Medical Center, Columbus, OH 43210, USA; Division of Endocrinology, Diabetes and Metabolism, College of Medicine, The Ohio State University Wexner Medical Center, Columbus, OH 43210, USA; Division of Endocrinology, Diabetes and Metabolism, College of Medicine, The Ohio State University Wexner Medical Center, Columbus, OH 43210, USA; The Ohio State University College of Medicine, Columbus, OH 43210, USA; Center for Biostatistics, The Ohio State University Wexner Medical Center, Columbus, OH 43210, USA; Division of Endocrinology, Diabetes and Metabolism, College of Medicine, The Ohio State University Wexner Medical Center, Columbus, OH 43210, USA

**Keywords:** continuous glucose monitor, type 2 diabetes, hospital

## Abstract

**Context:**

The post-hospitalization period is a vulnerable time for patients with type 2 diabetes (T2D).

**Objective:**

To assess usefulness of continuous glucose monitoring (CGM) for optimizing glucose levels and supporting medication use and behavioral change.

**Methods:**

We conducted a prospective, nonrandomized study of hospitalized adults with type 2 diabetes, HbA1c > 8%, and requiring ≥10 units of basal insulin daily. Participants received the Dexcom G6 and had follow-up visits at week 2, 4, 8, and 12 following discharge. The primary focus of analysis was change in HbA1c from 0 to 12 weeks. Secondary outcomes included CGM metrics, remote monitoring capability, and healthcare utilization.

**Results:**

Among 108 enrolled participants, 51% were monitored remotely, 79% had CGM data post-discharge, and 61% completed the 12-week visit. HbA1c (%) declined from 12% (interquartile range [IQR] 10%, 14%) to 8.2% (IQR 6.9%, 9.3%) (*P* < .0001). Time in glucose range 70 to 180 mg/dL (TIR) increased from 37% (IQR 17, 61) at 2 weeks to 43% (IQR 14, 86) at 12 weeks (*P* = .03). Among participants with endpoint HbA1c values, those with CGM data at all 4 visits, (44/60, 73%) had similar HbA1c, tended to be readmitted within 12 weeks less often (23% vs 50%, *P* = .06), and were more likely to have endocrinology follow-up (49% vs 6%, *P* = .003). Remote and manual monitoring groups had similar availability of CGM data, TIR, hypoglycemia, and healthcare utilization.

**Conclusion:**

Initiating CGM at hospital discharge was feasible, safe, and associated with significant glycemic improvement at 12 weeks. Additional studies are needed to optimize the implementation of CGM following discharge.

One-fourth of hospitalized patients have diabetes, and the frequency of hospitalization in persons with diabetes is anticipated to increase over time [1-3]. The risk of hospitalization increases with hyperglycemia [4, 5] and hypoglycemia [6], and despite treatment advances, glycated hemoglobin (HbA1c) levels have remained stagnant among persons with diabetes [7]. Post-discharge hyperglycemia may contribute to hospital readmission [8] and among patients with HbA1c above goal, hospitalization is associated with higher HbA1c at 1 year later [9]. The post-hospitalization period presents unique challenges including changes in patients’ daily routines and medications, competing priorities of comorbidities and other illnesses, and social determinants of health barriers. To help patients achieve glucose goals after hospitalization, the American Diabetes Association emphasizes the need for multifactorial discharge plans [10].

Glucose monitoring is a critical component of achieving glycemic control, allowing people with diabetes to adjust their diet and activity as well as medications. Continuous glucose monitoring (CGM) devices have become increasingly accurate and financially accessible. Meta-analyses of randomized controlled trials found that use of CGM is associated with reduced HbA1c when worn continuously [11-13] and epidemiologic studies have found that CGM is associated with reduced risk for hospitalization [14-16].

However, initiation of CGM requires significant resources, including obtaining insurance authorizations, device set-up and access to data, education on interpreting sensor values, alarms and alerts, and connecting to an appropriate provider to review data and provide feedback. Thus, the steps needed for optimal implementation of CGM at hospital discharge constitute a significant logistical barrier to widespread use. Furthermore, studies have shown that maintaining HbA1c benefit from CGM requires ongoing use [17-19], and personalized device education when initiating CGM is linked to better outcomes [19, 20]. Certified Diabetes Care and Education Specialists are in an ideal position to initiate CGM but they are not widely available in the hospital setting. Nevertheless, hospitalization presents a period of intense facetime with the healthcare system and an opportunity to intervene [21-24].

To date, few studies have examined the real-world effectiveness of post-discharge CGM. These studies have elucidated barriers to implementation and variation in adherence, but have been limited in size and scope [25-28]. A recent comprehensive review highlighted the need for studies investigating CGM upon hospital discharge and outlined barriers to deployment at time of discharge including regulatory, logistical, and technical, as well as strategies to overcome them [29]. To fill this gap in the literature, the current prospective nonrandomized (real world) study sought to determine the effectiveness of CGM following hospital discharge in a diverse cohort of patients with type 2 diabetes (T2D).

## Methods

### Design and Participants

This was a prospective, single-center, 24-week nonrandomized study. Inclusion criteria were hospitalized patients aged 18 years with T2D, HbA1c > 8.0% (64 mmol/mol) within 3 months before enrollment, requiring at least 10 units of basal insulin per day, able to provide informed consent and willing to attend study visits after discharge. Exclusion criteria included type 1 diabetes, incarceration, pregnancy, or need for discharge to skilled nursing facility. Participants were identified through daily screening of inpatient services throughout the institution and were enrolled between April 26, 2022, and Feb 16, 2024. This study was approved by the Ohio State University Institutional Review Board and all patients provided informed consent.

### Intervention

All participants wore the Dexcom G6 CGM (unblinded) throughout the 12-week follow-up period. All patients received education from trained personnel (clinical research coordinator) on its use according to manufacturer instructions. Specifically, participants received training on sensor insertion, pairing, and replacement (approximately 5 minutes). Participants were monitored for up to 30 minutes to ensure successful sensor initiation (often only a few minutes). Alerts were customized, but generally they were <70 mg/dL for hypoglycemia and >300 mg/dL for hyperglycemia. Participants also received training on interpretation of readings, alert thresholds and trend arrows, and when to perform fingerstick glucose measures (approximately 10 minutes). Patients also received printed materials including low literacy “Quick Tips,” skin care instructions, and links to additional online manufacturer resources (written and video). A clinic Clarity account was established for each patient with target ranges set up as 70 to 180 mg/dL (approximately 5 minutes).

If the patient had a compatible device, the Dexcom G6 app and Clarity apps were set up (up to 20 minutes depending on a number of factors, such as the need to reset passwords to access email or install an app). A sharecode was set up for 1 year and documented in the patient's electronic medical record. In participants without a compatible device, a separate receiver was provided, and data were downloaded manually at each study visit.

All patients received standard discharge instructions using the electronic medical record as per usual practice. The discharge regimen and all prescriptions and clinical follow-up were determined by the inpatient team with input from the diabetes consult service if requested. Hospital discharge is typically coordinated by a patient care resource manager who arranges follow-up prior to discharge. A discharge summary was sent to the primary provider per routine practice.

In addition, baseline assessments of social support (measured using the Multidimensional Scale of Perceived Social Support, MSPSS) [30], e-health literacy (E-health Literacy Scale, EHL) [31], and numeracy (Subjective Numeracy Scale-3, SNS-3) [32] were obtained prior to discharge.

### Follow-Up Visits

Four study follow-up visits occurred at weeks 2, 4, 8, and 12 following hospital discharge. Participants were contacted at prearranged times, with up to 3 attempts during the week of the encounter. In the event of failure to contact, a letter/email was sent and staff then reached out to the patient designated secondary contacts (up to 2 additional contacts). In the event of failure to contact, the medical records were monitored for up to 12 weeks following enrollment. Incentives were provided for completing follow-up HbA1c at the 12-week visit.

For all participants, study visits were conducted in-person at weeks 4 and 12. For persons with a compatible smartphone who were able to transmit data via the Clarity app, study visits at weeks 2 and 8 were conducted via telephone, but could be changed to in-person if additional supplies were needed or in case of barriers to CGM use that required re-training. For the remaining individuals, study visits at weeks 2 and 8 were in-person. At study visits, the study team confirmed CGM use and addressed any barriers. In addition, the team provided reminders to the patient regarding medication taking and instructions, as well as basal insulin titration, and encouraged hospital follow-up with usual care providers. Participants were encouraged to follow consistent carbohydrate meals. However, all other medication adjustments were left to the discretion of the usual diabetes provider. A follow-up HbA1c test was performed at 12 weeks.

### Communication With Usual Care Providers

Usual diabetes care providers (primary care or endocrinology, identified using the electronic medical record or via patient self-report) were notified of initial patient enrollment in the study using a standardized letter containing the patient's Clarity sharecode (if applicable) and instructions for accessing data. After each visit, the study team provided a letter with a summary of the CGM interpretation as well as any additional advice to the usual care provider.

### Data Collection

Data collection was conducted using REDCap. At enrollment, baseline data included demographics, education, employment, insurance information, past medical history, hospitalization characteristics, discharge medications, height, weight, and contact information. Post-discharge, the study coordinator solicited self-reported data including insulin dosing and diabetes medications, hypoglycemia, device-related concerns, and any healthcare utilization. CGM reports consisted of the ambulatory glucose profile from the previous 14 days at each study visit. Comorbidity was also assessed using the Charlson comorbidity index [33].

### Endpoints

The primary endpoint was change in % time in range (TIR) 70 to 180 mg/dL from week 2 to week 12 and the secondary endpoints were change in HbA1c from baseline to week 12, CGM time in ranges (%time >180 mg/dL, %time >300 mg/dL, %time <54 mg/dL, %time <70 mg/dL), %time with CGM data, and ability to use remote monitoring. However, due to more missing CGM data than expected, the primary focus of the paper is HbA1c change from baseline to week 12. HbA1c levels and health care utilization data were collected at study visits or extracted from the electronic medical record (when available) for participants with missing data. Exploratory endpoints included healthcare utilization data (emergency visits, hospitalizations, primary care or endocrinology visits).

### Sample Size

We estimated that a sample size of 80 individuals would yield 98% power to detect a 0.4% difference from a hypothesized mean 0.7% reduction in HbA1c, assuming a SD of 0.9%, and significance level of 0.05 [15]. We enrolled 100 individuals assuming a 20% loss to follow-up. An amendment to increase the total enrolled sample size to 120 individuals was approved after 1 year of enrollment due to greater than expected loss to follow-up.

### Analysis

In this report we assessed baseline characteristics and utilization of remote monitoring as well as its impact on clinical outcomes. We also compared characteristics between participants with and without consistent CGM wear (defined as having CGM data at all 4 study visits vs <4 visits with data) and its associations with clinical outcomes. Categorical variables were summarized as frequencies (n) and percentages (%) and compared using Chi-squared test or Fisher exact test as appropriate. Continuous variables were summarized as mean and SD and compared using 2-sample *t* test if they were normally distributed; non-normal distributions were summarized with median and interquartile range (IQR, 25th and 75th percentile) and compared using Wilcoxon rank sum test. Multivariable linear regression analysis was conducted to evaluate the association between consistent CGM wear and change in HbA1c from baseline to 12 weeks, adjusting for baseline HbA1c, marital status, and endocrinology follow-up status. We used all available data in the statistical analyses except where otherwise noted. We did not perform any data imputations for missing values. The statistical analysis was performed using R software (version 4.4.2; R Core Team, R Foundation for Statistical Computing, Vienna, Austria).

## Results

### Participants

**Figure 1.**
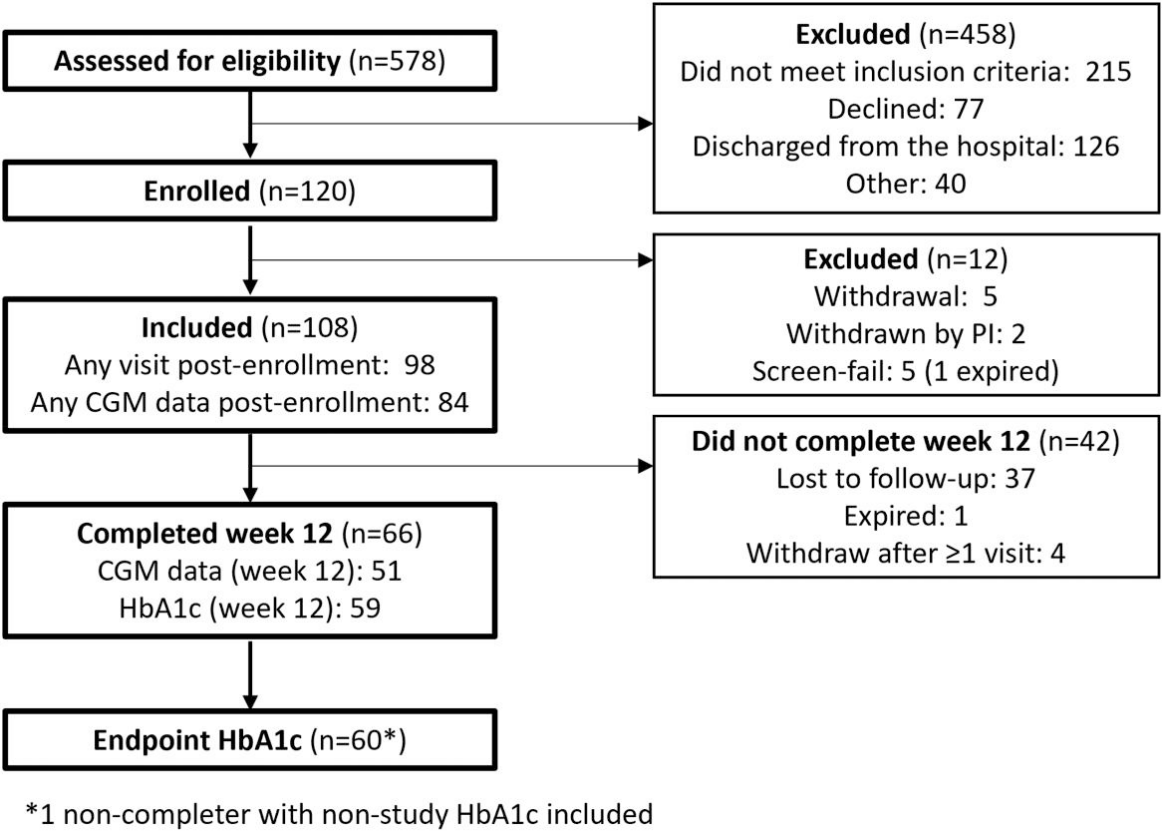
Participant flow diagram.

A total of 578 participants were assessed for eligibility and 120 were enrolled in the study (Fig. 1). Among persons excluded from the study, 215 (47%) did not meet inclusion criteria, 77 (17%) declined to participate, 126 (28%) were discharged from the hospital before enrollment, and 40 (9%) had other reasons. Among the 120 participants who signed consent, 5 (4%) were considered screen failures and 7 (5%) withdrew, leaving 108 subjects.

Characteristics of the 108 participants stratified by disposition are included in Tables 1 and 2. Overall 85 participants (79%) had CGM data at one or more study visits following hospital discharge while 66 (61%) completed the final 12-week study visit (completers). Completers and non-completers had similar baseline characteristics (mean age 52 [IQR 42, 58] vs 55 [IQR 47, 60] years; 56% vs 55% male; 56% vs 60% Black) and baseline HbA1c % (11.4 [IQR 10.0, 13.3] vs 11.7 [IQR 9.30, 12.4] *P* = .40). Completers had similar diabetes duration (15 [IQR 5, 21] vs 14[IQR 5, 20] years, *P* = .51) and 61% vs 69% were on insulin prior to admission (*P* = .37). Completers were similarly able to participate in remote monitoring (*P* = .52) but were more likely to have primary care provider follow-up (67% vs 26%, *P* < .001) and report provider review of CGM data (66% vs 20%, *P* < .001).

**Table 1.** Patient characteristics by disposition (all participants)

Characteristic	Overall N = 108*^a^*	Disposition		*P* value*^b^*
		Did not complete study n = 42*^1^*	Completed study n = 66*^1^*	
Age, years, mean (SD)	53 (46, 60)	55 (47, 60)	52 (42, 58)	.32
Gender, male	60 (56%)	23 (55%)	37 (56%)	.90
Race				.72
White	46 (43%)	17 (40%)	29 (44%)	
Black	62 (57%)	25 (60%)	37 (56%)	
Hispanic	2 (1.9%)	1 (2.4%)	1 (1.5%)	>.99
Married	36 (33%)	15 (36%)	21 (32%)	.68
Work status				.67
Unemployed	25 (23%)	12 (29%)	13 (20%)	
Disabled	21 (19%)	8 (19%)	13 (20%)	
Employed	45 (42%)	17 (40%)	28 (42%)	
Retired	17 (16%)	5 (12%)	12 (18%)	
Insurance				.15
None	10 (9.3%)	2 (4.8%)	8 (12%)	
Private	34 (31%)	10 (24%)	24 (36%)	
Medicare	28 (26%)	15 (36%)	13 (20%)	
Medicaid	36 (33%)	15 (36%)	21 (32%)	
Tobacco use, current	31 (29%)	13 (31%)	18 (27%)	.68
Diabetes duration, years	n = 106 15 (5, 20)	n = 41 14 (5, 20)	n = 65 15 (5, 21)	.51
Pre-admission insulin therapy	69 (64%)	29 (69%)	40 (61%)	.37
Body mass index (kg/m^2^)	33 (28, 40)	33 (29, 41)	32 (28, 40)	.81
eGFR, mL/min/1.73 m^2^	82 (54, 99)	76 (43, 103)	83 (65, 98)	.40
Admit reason				.13
Diabetes	26 (24%)	12 (29%)	14 (21%)	
Infectious disease	37 (34%)	16 (38%)	21 (32%)	
Cardiovascular	27 (25%)	6 (14%)	21 (32%)	
Gastrointestinal	6 (5.6%)	1 (2.4%)	5 (7.6%)	
Other	12 (11%)	7 (17%)	5 (7.6%)	
Charlson Comorbidity Index	4.00 (2.00, 6.50)	3.50 (1.00, 7.00)	4.00 (2.00, 6.00)	.98
Length of hospital stay, days	5.0 (3.0, 7.0)	6.0 (3.0, 8.0)	5.0 (3.0, 7.0)	.22
Diabetes consult	69 (64%)	25 (60%)	44 (67%)	.45
Remote monitoring				.52
App on phone	55 (51%)	21 (50%)	34 (52%)	
Reader/no smartphone	23 (21%)	11 (26%)	12 (18%)	
Reader/phone not compatible	27 (25%)	10 (24%)	17 (26%)	
Other	3 (2.8%)	0 (0%)	3 (4.5%)	
Baseline HbA1c	11.6 (9.65, 12.9)	11.7 (9.30, 12.4)	11.4 (10.0, 13.3)	.40

^a^Median (Q1, Q3); n (%), unless otherwise specified. ^*b*^Wilcoxon rank sum test; Fisher's exact test.

**Table 2.** Other on-study data stratified by disposition (all participants)

Characteristic	Overall N = 108*^a^*	Disposition		*P* value*^b^*
		Did not complete study n = 42*^a^*	Completed study n = 66*^a^*	
Insulin use at baseline, units				
Total daily dose	N = 103 54 (30, 87)	n = 40 48 (28, 85)	n = 63 56 (33, 90)	.53
Basal insulin	N = 108 36 (20, 56)	n = 38 36 (20, 45)	n = 62 36 (20, 60)	.22
Sensor events				
Any sensor failure	N = 84 15 (18%)	n = 19 2 (11%)	n = 65 13 (20%)	.50
Any bleeding	N = 84 9 (11%)	n = 19 1 (5.3%)	n = 65 8 (12%)	.68
Any pain	N = 84 5 (6.0%)	n = 19 0 (0%)	n = 65 5 (7.7%)	.58
Any infection	N = 84 0 (0%)	n = 19 0 (0%)	n = 65 0 (0%)	—
Any pruritus	N = 84 11 (13%)	n = 19 0 (0%)	n = 65 11 (17%)	.06
Any other sensor issue	N = 85 6 (7.1%)	n = 20 4 (20%)	n = 65 2 (3.1%)	.03
Usual provider-reviewed data, patient-reported	N = 90 48 (53%)	n = 25 5 (20%)	n = 65 43 (66%)	<.001
Healthcare utilization				
Any Emergency department use	N = 92 26 (28%)	n = 26 8 (31%)	n = 66 18 (27%)	.74
Any readmission	N = 92 26 (28%)	n = 26 5 (19%)	n = 66 21 (32%)	.23
Any primary care provider follow-up	N = 93 51 (55%)	n = 27 7 (26%)	n = 66 44 (67%)	<.001
Any endocrinology follow-up	N = 92 29 (32%)	n = 26 5 (19%)	n = 66 24 (36%)	.11
Any provider follow-up	N = 92 62 (67%)	n = 26 11 (42%)	n = 66 51 (77%)	.001

^a^Median (Q1, Q3); n (%).

^b^Wilcoxon rank sum test; Fisher's exact test; Pearson's Chi-squared test.

### Device Connection

Overall, 55 (51%) of participants had a compatible smartphone and agreed to be monitored remotely (Table 3). Of the remaining 53 participants, 25% had a smartphone that was not compatible, 21% did not have a smartphone, and 3% had other reasons (no email, personal preference). Participants who were monitored remotely were younger (50 [41, 57] vs 58 [IQR 49, 64] years, *P *< .001), more likely to be male (66% vs 41%, *P* = .03), and more likely to be employed (56% vs 26%) (*P* < .001, ANOVA for difference across employment groups, Table 3). Participants using remote monitoring had similar baseline social support (measured using the Multidimensional Perceived Social Support Scale), e-health literacy (E-health Literacy Scale), and numeracy (Subjective Numeracy Scale-3) as those requiring all in-person visits.

**Table 3.** Participant characteristics by remote monitoring

	Overall N = 108*^a^*	No remote monitoring N = 53*^a^*	Remote monitoring N = 55*^a^*	*P* value*^b^*
**Baseline**				
Age, years	53 (46, 60)	58 (49, 64)	50 (41, 57)	<.001*^c^*
Male	60 (56%)	35 (66%)	25 (41%)	.03
Race				.35
White	46 (43%)	25 (47%)	21 (38%)	
Black	62 (57%)	28 (53%)	34 (62%)	
Hispanic	2 (1.9%)	0 (0%)	2 (3.6%)	.50
Education > high school	N = 107 28 (26%)	N = 52 14 (27%)	N = 55 14 (25%)	.86
Married	36 (33%)	21 (40%)	15 (27%)	.17
Employment Status				<.001
Unemployed	25 (23%)	10 (19%)	15 (27%)	
Disabled	21 (19%)	16 (30%)	5 (9.1%)	
Employed	45 (42%)	14 (26%)	31 (56%)	
Retired	17 (16%)	13 (25%)	4 (7.3%)	
Insurance				.19
None	10 (9%)	3 (5.7%)	7 (13%)	
Private	34 (32%)	14 (26%)	20 (36%)	
Medicare	28 (26%)	18 (34%)	10 (18%)	
Medicaid	36 (33%)	18 (34%)	18 (33%)	
Diabetes duration, years	15 (5, 20)	15 (7, 23)	14 (5, 20)	.16
Diabetes consult	58 (64%)	35 (66%)	23 (62%)	.69
MSPSS	5.5 (3.9, 6.7)	5.5 (3.8, 6.7)	5.2 (4.1, 6.7)	.96
E-health Literacy Scale	39 (35, 41)	38 (34, 41)	40 (35, 42)	.54
Subjective Numeracy Scale-3	12 (8, 15)	11 (7, 15)	13 (9, 16)	.16
Baseline HbA1c	11.55 (9.6, 12.9)	11.7 (9.50, 13.2)	11.4 (9.90, 12.5)	.59

Abbreviations: HbA1c, glycated hemoglobin; MSPSS, Multidimensional Scale of Perceived Social Support.

^a^Median (Q1, Q3); n (%), unless otherwise stated.

^b^Wilcoxon rank sum test; Fisher's exact test/Wilcoxon rank sum exact test, unless otherwise stated.

^c^ *t* test.

CGM data availability at study visits was similar between participants with and without remote monitoring. A comparable proportion had any CGM data available (87% vs 74%) and CGM data at all 4 visits (51% vs 43%) (Table 4). Healthcare utilization was also similar, with 67% reporting any provider follow-up in both groups. Hospital readmission was 22% vs 35% in remotely vs not remotely monitored groups (*P* = .25). The %TIR was also similar across groups at 12 weeks (55% [IQR 17, 89] vs 37% [14, 79]), *P* = .36). HbA1c % decreased similarly across groups (−2.8 [IQR −4.7, −0.51] vs −3.9 [IQR −5.1, −1.1], *P* = .42).

**Table 4.** Outcomes by monitoring approach

	Overall N = 108*^a^*	No remote monitoring N = 53*^a^*	Remote monitoring N = 55*^a^*	*P* value*^b^*
Change in HbA1c	N = 60 −3.3 (−4.7, −0.75)	N = 29 −3.9 (−4.71, −1.4)	N = 31 −2.8 (−4.7, −0.51)	.42
Availability of CGM data				
Week 2, in-person	78 (72%)	34 (64%)	44 (80%)	.07
Week 4, remote	71 (66%)	33 (62%)	38 (69%)	.46
Week 8, remote	67 (62%)	31 (58%)	36 (65%)	.46
Week 12, in-person	59 (55%)	30 (57%)	29 (53%)	.69
Provider-reviewed data, patient-reported	N = 89 48 (55%)	N = 40 24 (59%)	N = 49 24 (50%)	.40
CGM metrics Week 12	N = 59	N = 30	N = 29	
Mean glucose, median	198 (139, 253)	203 (146, 269)	179 (136, 232)	.25
Glucose variability (CV, %)	33.6	33.2	33.7	−
%>250 mg/dL	17 (1, 49)	25.5 (2, 56)	14 (0, 35)	.16
%>180 mg/dL	57 (10, 85)	63 (18, 86)	44 (9, 83)	.38
%TIR week 12	43 (14, 86)	37 (14, 80)	55 (17, 89)	.36
%<70 mg/dL	0 (0, 1)	0.2 (0, 1.25)	0 (0, 1)	.56
%<54 mg/dL	0 (0, 1)	0 (0, 1)	0 (0, 0.5)	.57
Health care utilization	N = 92	N = 43	N = 49	
Hospital readmission	26 (28%)	15 (35%)	11 (22%)	.19
Any PCP follow-up*^c^*	51 (55%)	24 (55%)	27 (55%)	.96
Any endocrinology follow-up	29 (32%)	14 (33%)	15 (31%)	.84
Any provider follow-up	62 (67%)	29 (67%)	33 (67%)	.99

Abbreviations: CGM, continuous glucose monitor; CV, coefficient of variation of mean glucose (%); HbA1c, glycated hemoglobin; PCP, primary care provider; TIR, time in range 70-180 mg/dL.

^a^Median (Q1, Q3); n (%), unless otherwise stated.

^b^Wilcoxon rank sum test; Fisher's exact test/Wilcoxon rank sum exact test, unless otherwise stated.

^c^N = 44 in the No Remote Monitoring Group and N = 93 overall.

### HbA1c

Among the 66 participants who completed the 12-week study visit, 59 (89%) had HbA1c values. One participant who was lost to follow-up had a non-study week-12 HbA1c measurement and was included in this analysis. Among the 60 participants assessed for change in HbA1c (the HbA1c cohort), 44 (73%) provided CGM data at all 4 study visits (consistent CGM use). Characteristics of the HbA1c cohort (stratified by consistent vs inconsistent CGM use) are shown in Table 5. Mean age was 53 ± 10 years, 55% of participants identified as male, 55% as Black, and 1 as Hispanic. Approximately half of participants were insured by Medicare or Medicaid, one-third were insured privately, and the remaining 12% were not insured.

**Table 5.** Patient characteristics by CGM use (among N = 60 subjects included in HbA1c analysis)

Characteristic	Overall N = 60*^a^*	CGM use		*P* value*^b^*
		<4 visits (inconsistent use) n = 16*^a^*	4 visits (consistent use) n = 44*^a^*	
Age, years, mean (SD)	54 (48, 61)	59 (56, 63)	51 (46, 59)	.02*^c^*
Male	33 (55%)	11 (69%)	22 (50%)	.20
Race				.64
White	27 (45%)	8 (50%)	19 (43%)	
Black	33 (55%)	8 (50%)	25 (57%)	
Hispanic	1 (1.7%)	0 (0%)	1 (2.3%)	>.99
Education > high school	16 (27%)	5 (31%)	11 (26%)	.75
Married	19 (32%)	6 (38%)	13 (30%)	.56
Employment status				.40
Unemployed	11 (18%)	3 (19%)	8 (18%)	
Disabled	11 (18%)	5 (31%)	6 (14%)	
Employed	27 (45%)	5 (31%)	22 (50%)	
Retired	11 (18%)	3 (19%)	8 (18%)	
Insurance				.01
None	7 (12%)	0 (0%)	7 (15%)	
Private	21 (35%)	2 (14%)	19 (41%)	
Medicare	14 (23%)	5 (31%)	9 (20%)	
Medicaid	18 (30%)	9 (56%)	9 (20%)	
Tobacco use, current	18 (30%)	5 (31%)	13 (30%)	>.99
Diabetes duration, years	15 (5, 22) N = 59	18 (13, 22) n = 15	14 (5, 22) n = 44	.12
Pre-admission insulin therapy	38 (63%)	10 (63%)	28 (64%)	.94
BMI, kg/m^2^	33 (28, 40)	31 (26, 37)	34 (28, 40)	.38
eGFR, mL/min/1.73m^2^	83 (63, 98)	82 (61, 95)	84 (65, 102)	.58
Length of hospital stay, days	5.0 (3.0, 7.0)	6 (3.5, 7.5)	4.5 (3.0, 6.0)	.15
Admission reason				—
Diabetes	11 (18%)	5 (31%)	6 (14%)	
Infectious disease	21 (35%)	4 (25%)	17 (39%)	
Cardiovascular	18 (30%)	4 (25%)	14 (32%)	
Gastrointestinal	5 (8.3%)	3 (19%)	2 (4.6%)	
Other	5 (8.3%)	0 (0%)	5 (11%)	
Diabetes consult	40 (67%)	9 (56%)	31 (70%)	.30
MSPSS	5.4 (3.5, 6.7)	5.96 (4.4, 6.8)	5.1 (3.2, 6.6)	.21
E-health Literacy Scale	39 (33, 42)	38 (33, 42)	39 (36, 42)	.63
Subjective Numeracy Scale-2	12.0 (7.5, 15.0)	12.0 (8.5, 15.0)	11.0 (6.5, 15.0)	.59
Remote monitoring, yes	31 (52%)	6 (38%)	25 (57%)	.20
App on phone	31 (52%)	6 (38%)	25 (57%)	
Reader/no smartphone	11 (18%)	6 (38%)	5 (11%)	
Reader/phone not compatible	15 (25%)	4 (25%)	11 (25%)	
No email	1 (1.7%)	0 (0%)	1 (2.3%)	
Other	2 (3.3%)	0 (0%)	2 (4.5%)	
Charlson Comorbidity Index	4.0 (2.0, 5.5)	4.5 (2.5, 6.5)	3.0 (2.0, 5.0)	.12
Baseline HbA1c	11.7 (10, 13.5)	11.4 (9.45, 13.6)	11.8 (10.4, 13.4)	.65
HbA1c week 12	8.20 (6.85, 9.30)	8.85 (7.50, 9.70)	7.70 (6.80, 9.05)	.21
Change in HbA1c	−3.25 (−4.70, −0.75)	−2.00 (−4.20, −0.15)	−3.70 (−5.05, −0.95)	.21

Abbreviations: BMI, body mass index; HbA1c, glycated hemoglobin; MSPSS, Multidimensional Scale of Perceived Social Support.

^a^Median (Q1, Q3); n (%), unless otherwise stated.

^b^Wilcoxon rank sum test; Fisher's exact test/Wilcoxon rank sum exact test, unless otherwise stated.

^c^ *t* test.

Overall, the HbA1c decreased from 11.7% (IQR 10, 13.5), to 8.2% (IQR 6.9, 9.3) (*P* < .0001) and there was a similar reduction in consistent CGM users compared to inconsistent users (−3.7 [IQR −5.1, −.95] vs −2 [IQR −4.2, −0.2], *P* = .21). In multivariable linear regression analysis (Table 6), after adjusting for baseline HbA1c, marital status, and any endocrine follow-up visit, consistent CGM use (4 visits with CGM data) was not associated with change in HbA1c from baseline to 12 weeks. Of note marital status was associated with greater reduction in HbA1c in this model but endocrine follow-up was not.

**Table 6.** Multivariable linear regression model—change in HbA1c (N = 60)

	Estimate	Lower 95% CI	Upper 95% CI	*P* value
Consistent CGM use (4 visits)	−0.73	−2.02	0.56	.26
Baseline HbA1c	−0.58	−0.85	−0.31	<.001
Marital status (married)	−1.38	−2.55	−0.21	.02
Any endocrine follow-up (yes)	−0.69	−1.99	0.61	.29

Abbreviations: CGM, continuous glucose monitoring; HbA1c, glycated hemoglobin.

### CGM Data

**Figure 2.**
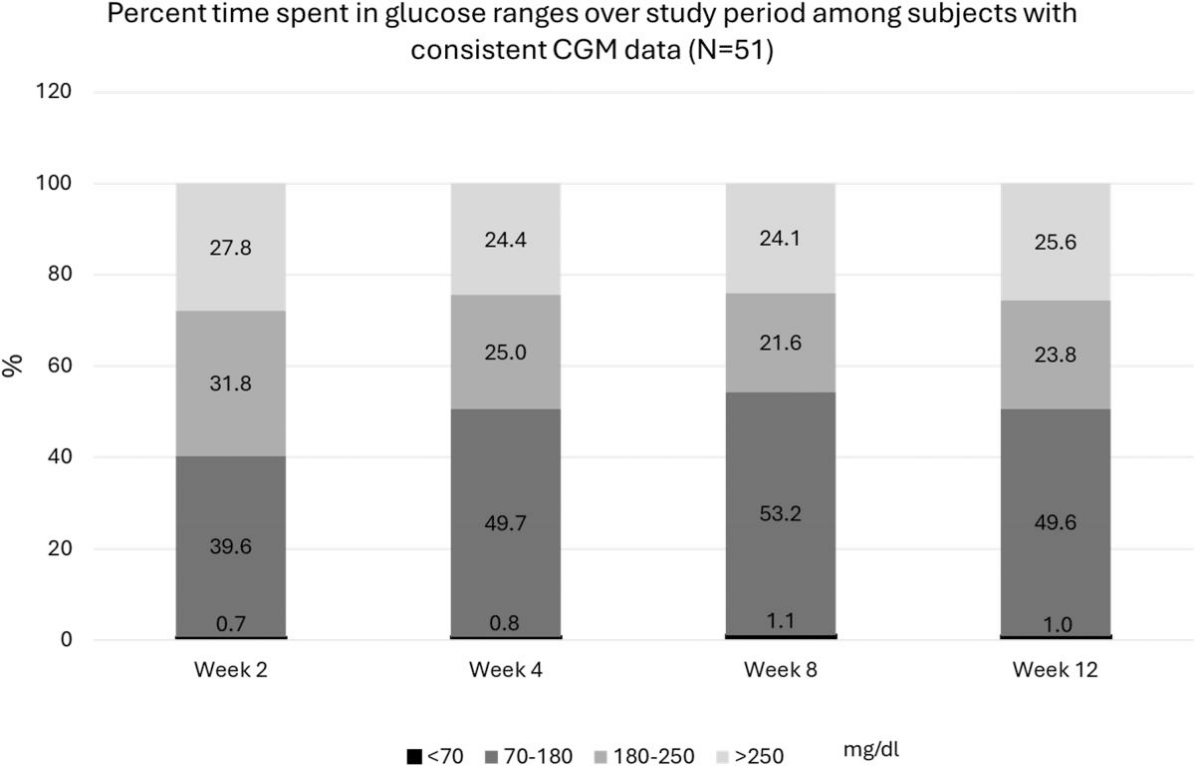
CGM % time in ranges. Participants included had continuous CGM data available at all fo4ur time points. Abbreviations: CGM, continuous glucose monitoring; TIR, time in range.

Percent time spent in glucose ranges by all participants is shown in Table 7. Wear time was > 95% at all time points for those with available data. TIR improved from 33% (IQR 17, 64) to 48% (IQR 4.7, 82) from 2 weeks to 12 weeks, *P* = .03 (paired *t* test, N = 54). The % time below 70 mg/dL was under 1% at all time points. Figure 2 depicts time in blood glucose ranges and mean glucose among all subjects with consistent CGM use (N = 51).

**Table 7.** CGM percent time in glucose ranges

All participants (N = 108)	% <54 mg/dL	% < 70 mg/dL	% 70-180 mg/dL	% > 180	% > 250 mg/dL
Week 2, n = 79	0.0 (0.0, 0.3)	0.0 (0.0, 1.0)	37 (17, 61)	63 (39, 83)	19 (6, 48)
Week 4, n= 71	0.0 (0.0, 0.2)	0.1 (0.0, 1.0)	49 (25, 78)	51 (21, 75)	9 (2, 33)
Week 8, n = 67	0.0 (0.0, 1)	0.4 (0.0, 1.0)	47 (24, 86)	52 (14, 75)	11.4 (1, 32)
Week 12, n = 59	0.0 (0.0, 1.0)	0.0 (.00, 1.0)	43 (14, 86)	57 (10, 85)	17 (1, 49)

Data reported as median (25%, 75%). All participants with available data were included at each time point, regardless of disposition.

Abbreviation: CGM, continuous glucose monitor.

### Other Study Outcomes

Other data from the HbA1c cohort, stratified by consistent vs inconsistent CGM use, are shown in Table 8. The HbA1c group included 44 participants with consistent CGM use and 16 with inconsistent CGM use. Insulin dose and non-insulin medications were similar throughout the study, with 27% of participants reporting a new glucose-lowering medication, and the most common non-insulin medications at 12 weeks being metformin (45%), sodium-glucose cotransporter type 2 inhibitor (SGLT2i) (27%) or glucagon-like peptide 1 (GLP-1) based therapy (33%). In this group, 20% of patients reported needing to replace the sensor prior to 10 days, 12% reported bleeding (all minor), 9% sensor pain, and 0% infection. There were no serious adverse events.

**Table 8.** Other on-study data stratified by CGM use (A1c Cohort)

		CGM use		
Characteristic	Overall N = 60*^a^*	Inconsistent use n = 16*^a^*	Consistent use n = 44*^a^*	*P* value*^b^*
Insulin use				
Total daily dose (units)				
Baseline	N = 58 55 (30, 87)	n = 16 38 (29, 69)	n = 42 58 (37, 87)	0.33
Week 12	N = 53 52 (29, 81)	n = 13 52 (36, 70)	n = 40 50 (29, 84)	0.88
Change, baseline to week 12	N = 53 0 (−12, 6)	n = 13 0 (−12, 12)	n = 40 −1 (−12, 6)	0.46
Basal insulin (units)				
Baseline	N = 57 35 (20, 60)	n = 16 38 (20, 64)	n = 41 34 (20, 56)	0.86
Week 12	N = 48 40 (23, 63)	n = 12 46 (27, 69)	n = 36 39 (23, 58)	.56
Change (baseline to week 12)	N = 48 0 (−2, 2)	n = 12 0 (0, 5)	n = 36 0 (−2, 2)	.55
Non-insulin medications baseline				
Thiazolidinedione	1 (1.7%)	0 (0)	1 (2.3%)	>.99
Metformin	26 (43%)	6 (38%)	20 (46%)	.58
Sulfonylurea	3 (5%)	0 (0)	3 (6.8%)	.56
SGLT2i	17 (28%)	6 (38%)	11 (25%)	.35
DPP4i	3 (5%)	0 (0)	3 (6.8%)	.56
GLP1RA	16 (27%)	5 (31%)	11 (25%)	.74
None	5 (8.3%)	2 (13%)	3 (6.8%)	.60
Non-insulin medications, week 12				
Thiazolidinedione	1 (1.7%)	0 (0)	1 (2.3%)	>.99
Metformin	27 (45%)	6 (38%)	21 (48%)	.48
Sulfonylurea	4 (6.7%)	2 (13%)	2 (4.5%)	.29
SGLT2i	16 (27%)	7 (44%)	9 (21%)	.10
DPP4i	5 (8.3%)	1 (6.3%)	4 (9.1%)	>.99
GLP1RA	20 (33%)	7 (44%)	13 (30%)	.30
Other	2 (3.3%)	0 (0)	2 (4.5%)	>.99
None	5 (8.3%)	0 (0)	5 (11%)	.31
Any new non-insulin medication	16 (27%)	7 (44%)	21 (21%)	.10
Sensor events	N = 59	N = 15	N = 44	
Sensor failure	12 (20%)	3 (20%)	9 (20%)	>.99
Bleeding	7 (12%)	0 (0%)	7 (16%)	.17
Pain	5 (8.5%)	0 (0%)	5 (11%)	.32
Infection	0 (0%)	0 (0%)	0 (0%)	
Pruritus	11 (19%)	2 (13%)	9 (21%)	.71
Other	3 (5.1%)	3 (20%)	0 (0%)	.01
Level 3 hypoglycemia	3 (5%)	1 (6%)	2 (5%)	>.99
Usual provider-reviewed data (patient-reported)	N = 59 40 (68%)	n = 15 8 (53%)	n = 44 32 (73%)	.21
Healthcare utilization				
Emergency department visit	15 (25%)	5 (31%)	10 (24%)	.52
Readmission	18 (30%)	8 (50%)	10 (23%)	.06
Any primary care provider follow-up	38 (63%)	8 (50%)	30 (68%)	.20
Any endocrinology follow-up	22 (37%)	1 (6.3%)	21 (48%)	.003
Any provider follow-up	45 (75%)	9 (56%)	36 (82%)	.09

Abbreviations: CGM, continuous glucose monitoring; DPP4i, dipeptidyl peptidase-4 inhibitor; GLP1RA, glucagon-like peptide-1 receptor agonist; n, number of participants in subgroup; N, total number of participants in analysis; SGLT2i, sodium-glucose cotransporter-2 inhibitor.

^a^Median (Q1, Q3); n (%).

^b^Wilcoxon rank sum test; Fisher's exact test; Pearson's Chi-squared test.

In the HbA1c cohort, severe (level 3) hypoglycemia occurred in 5% vs 6% of consistent vs inconsistent CGM users respectively (*P* > .99). In addition, consistent CGM users had less than half the readmission rate of those with inconsistent CGM use (23% vs 50%), with borderline statistical significance (*P* = .06). Among participants with consistent CGM use, 20 (49%) had any endocrinology follow-up visit compared to 1 (6%) of those with inconsistent CGM use (*P* = .003). There were no other notable differences in characteristics between consistent vs inconsistent CGM use.

## Discussion

This study demonstrated that integrating CGM at hospital discharge within a referral-based academic health system is feasible; most patients successfully downloaded or transmitted CGM data at least once. CGM was well tolerated in this medically complicated population and significant hypoglycemia was uncommon. Importantly, participants achieved clinically meaningful HbA1c reductions, with sustained improvements in TIR in those with ongoing use.

These findings are consistent with and extend the findings of prior research. A pilot randomized clinical trial (N = 100) found that initiating intermittently scanned FreeStyle Libre 2 CGM at discharge, compared to capillary point of care (POC) glucose testing plus blinded CGM, was associated with nonsignificant trends toward improved TIR (70-180 mg/dL) and time above range (>180 and >250 mg/dL), and decreased prandial insulin requirements [34]. Of note, only 52% and 56% of the CGM and POC groups had available CGM data at the 12-week follow-up, similar to our study cohort. Additionally, a small study (N = 20) concluded that CGM initiation at hospital discharge was both feasible and beneficial. However, as with the present study, device incompatibility issues limited the ability to perform remote monitoring [27]. Another small randomized study (N = 30) utilizing the FreeStyle Libre 2 found that CGM use at emergency visit discharge was a practical approach for engaging high-risk patients with limited access to consistent medical care, although only 9 of 16 (56%) participants in the CGM group attended follow-up and had available data [35]. The study also showed a trend toward improved HbA1c at 3 months.

While 27% of participants started a new medication during study follow-up, insulin doses remained stable throughout the study. Thus, the dramatic reduction in HbA1c suggests that glycemic improvements may be driven by changes in insulin doses at the time of hospital discharge or in non-insulin medications after discharge. However, the improvement in CGM TIR from week 2 to week 12, despite stable insulin doses, also supports the role of behavioral adaptation on overall glycemia. This aligns with research from ambulatory settings, which demonstrates that CGM improves glycemic outcomes without substantial change in concomitant medications [6], and enhances diabetes self-management by providing real-time glucose feedback and promoting both short-term and long-term behavioral adjustments [36].

Despite these benefits, barriers to CGM implementation were evident, similar to those highlighted in a recent comprehensive review [29]. One such barrier was diabetes and device education. In our study, patients were trained on CGM use by a clinical research coordinator without formal medical training, rather than a Certified Diabetes Care and Education Specialist. Training sessions occurred just prior to discharge, lasted less than 20 minutes for patients with a receiver, and could reasonably be integrated into standard bedside discharge teaching. Notably, more than half of participants had a high school education or less. Diabetes-specific knowledge was not measured, but intensive training may have resulted in better outcomes or persistent use. Nevertheless, the glycemic benefits achieved without intensive education illustrate the potential for broader adoption as diabetes education is not reimbursable in the hospital and many hospital systems lack this resource [37].

Another barrier was that nearly half of participants (48%) lacked a compatible smartphone device and could not participate in remote monitoring activities. Although 91% of US adults now own a smartphone, most CGM systems remain compatible with limited range of smartphone brands and models—restricting accessibility for many users [38]. Participants without a compatible smartphone were older, more likely to be male, and less likely to be employed. The additional time required for smart device set-up varies widely in part depending on the need to reset passwords to install. On the other hand, the availability of CGM data and glycemic outcomes were similar regardless of monitoring strategy, highlighting the potential for telehealth or remote monitoring programs to expand access to diabetes care where feasible. Healthcare systems could also earn additional savings through improvement in Healthcare Effectiveness Data and Information Set measures, which now recognize the Glucose Management Indicator as an alternate to HbA1c [39]. Integration of CGM system metrics within the electronic medical record could more easily facilitate data capture. These data are similar to another study using the Freestyle Libre 2 in which 90 of 198 participants (45%) were willing and able to participate in remote monitoring [27].

While postponing initiation of CGM until after discharge ensures that logistical barriers such as prior authorizations are addressed and training is initiated, there are additional post-discharge hurdles such as access to care and competing priorities related to acute illness to consider [40]. Consistent with this is the observation that patients with specialty visit follow-up but not primary care follow-up were more likely to demonstrate consistent post-discharge CGM use. Disparities in use of CGM are well described in the ambulatory setting, and this is of particular concern in our study population in which 57% identified as Black, 19% were on disability, and 33% were on Medicaid [41]. As we did not observe significant differences in study completion among race or insurance categories, it is possible that initiating CGM at discharge may help address socioeconomic disparities in CGM access. Furthermore, CGM initiation during hospitalization could allow patients more time to orient themselves to the device under the general supervision of the healthcare team. In patients with newly diagnosed type 1 diabetes, initiating CGM soon after diagnosis results in higher sustained use, lower HbA1c levels, greater TIR, and lower emergency visits, compared to delayed initiation [42-44].

In our study, the frequency of timely post-hospital follow-up was lower than desired but consistent with prior research [45-47], suggesting that additional strategies are needed to ensure connection with a provider for CGM feedback and medication adjustments. In an observational study of 90 patients discharged on CGM with remote monitoring, technical barriers post-discharge included sensor application, device maintenance, and data interpretation [25]. Despite these challenges, 76% had their follow-up plans altered based on sensor data, demonstrating the importance of structured post-discharge support, and over half of those who were able to participate in remote monitoring were candidates for “case conferencing,” in which a diabetes specialist provided advice in place of a specialty referral. Additionally, patient navigators or community health workers could provide essential support in bridging the transition from hospital to home.

There were several limitations to this study. This was a nonrandomized study, as the goal was to examine the impact of CGM implementation following hospital discharge in a real-world population. Thus, improvements in HbA1c or TIR could not be definitively attributed to CGM alone and are likely multifactorial. Despite multiple methods of maintaining contact, there was significant loss to follow-up, which limited statistical power to assess outcomes, and in particular to stratify outcomes by CGM use. Moreover, we cannot assess whether participants who did not complete the study were using CGM. However, participants who completed the study had similar baseline characteristics as those who did not, suggesting the applicability to the broader population as a whole. Medication adjustments were not standardized and were left to provider discretion, which may have influenced the results. Lastly, this study did not address broader barriers to successful transitions of care, such as clinical inertia, mental health, physical disability, literacy, financial hardship, or lack of transportation, all of which can impact diabetes outcomes [10].

In conclusion, the study demonstrated that the implementation of CGM at hospital discharge is feasible and is associated with significant improvements in HbA1c and time in range, regardless of monitoring strategy (in-person vs remote). However significant barriers need to be addressed to achieve the optimal outcomes reported in other ambulatory settings, including device-related issues, such as availability of training and technical issues, and access to diabetes specialty follow-up care. The data indicate a critical need for studies evaluating potential implementation strategies in the post-discharge period.

## Data Availability

Some or all datasets generated during and/or analyzed during the current study are not publicly available but are available from the corresponding author on reasonable request.

## Clinical Trial Information

ClinicalTrials.gov identification no. NCT05360056.

## Abbreviations

CGM: continuous glucose monitoring
HbA1c: glycated hemoglobin
IQR: interquartile range
T2D: type 2 diabetes
TIR: time in range

## References

[1] Rubens M, Ramamoorthy V, Saxena A, McGranaghan P, McCormack-Granja E. Recent trends in diabetes-associated hospitalizations in the United States. J Clin Med. 2022;11(22):6636. 36431114 10.3390/jcm11226636 PMC9698503

[2] Centers for Disease Control and Prevention . *National Diabetes Statistics Report*. Atlanta, GA: US Department of Health and Human Services; 2024. Accessed 8 November 2025. https://www.cdc.gov/diabetes/php/data-research/index.html

[3] Zhang Y, Bullard KM, Imperatore G, Holliday CS, Benoit SR. Proportions and trends of adult hospitalizations with diabetes, United States, 2000-2018. Diabetes Res Clin Pract. 2022;187:109862. 35367522 10.1016/j.diabres.2022.109862 PMC11301745

[4] Li TC, Kardia SLR, Li CI, et al Glycemic control paradox: poor glycemic control associated with higher one-year and eight-year risks of all-cause hospitalization but lower one-year risk of hypoglycemia in patients with type 2 diabetes. Metabolism. 2015;64(9):1013‐1021. 26026367 10.1016/j.metabol.2015.05.004

[5] Mata-Cases M, Rodríguez-Sánchez B, Mauricio D, et al The association between poor glycemic control and health care costs in people with diabetes: a population-based study. Diabetes Care. 2020;43(4):751‐758. 32029636 10.2337/dc19-0573

[6] American Diabetes Association Professional Practice Committee . 6. glycemic goals and hypoglycemia: standards of care in diabetes-2025. Diabetes Care. 2025;48(Supplement_1):S128‐S145. 39651981 10.2337/dc25-S006 PMC11635034

[7] American Diabetes Association Professional Practice Committee . 1. improving care and promoting health in populations: standards of care in diabetes—2025. Diabetes Care. 2025;48(Supplement_1):S14‐S26. 39651974 10.2337/dc25-S001 PMC11635030

[8] Rubin DJ. Hospital readmission of patients with diabetes. Curr Diab Rep. 2015;15(4):17. 25712258 10.1007/s11892-015-0584-7

[9] Wei NJ, Grant RW, Nathan DM, Wexler DJ. Effect of hospital admission on glycemic control 1 year after discharge. Endocr Pract Off J Am Coll Endocrinol Am Assoc Clin Endocrinol. 2012;18(4):456‐463. 10.4158/EP11309.OR PMC3746561 22805110

[10] American Diabetes Association Professional Practice Committee . 16. Diabetes care in the hospital: standards of care in diabetes—2025. Diabetes Care. 2025;48(Supplement_1):S321‐S334. 39651972 10.2337/dc25-S016 PMC11635037

[11] Jancev M, Vissers TACM, Visseren FLJ, et al Continuous glucose monitoring in adults with type 2 diabetes: a systematic review and meta-analysis. Diabetologia. 2024;67(5):798‐810. 38363342 10.1007/s00125-024-06107-6 PMC10954850

[12] Seidu S, Kunutsor SK, Ajjan RA, Choudhary P. Efficacy and safety of continuous glucose monitoring and intermittently scanned continuous glucose monitoring in patients with type 2 diabetes: a systematic review and meta-analysis of interventional evidence. Diabetes Care. 2024;47(1):169‐179. 38117991 10.2337/dc23-1520

[13] Kieu A, King J, Govender RD, Östlundh L. The benefits of utilizing continuous glucose monitoring of diabetes Mellitus in primary care: a systematic review. J Diabetes Sci Technol. 2023;17(3):762‐774. 35100891 10.1177/19322968211070855 PMC10210096

[14] Guerci B, Roussel R, Levrat-Guillen F, et al Important decrease in hospitalizations for acute diabetes events following FreeStyle libre system initiation in people with type 2 diabetes on basal insulin therapy in France. Diabetes Technol Ther. 2023;25(1):20‐30. 36094418 10.1089/dia.2022.0271

[15] Roussel R, Riveline JP, Vicaut E, et al Important drop in rate of acute diabetes complications in people with type 1 or type 2 diabetes after initiation of flash glucose monitoring in France: the RELIEF study. Diabetes Care. 2021;44(6):1368‐1376. 33879536 10.2337/dc20-1690 PMC8247513

[16] Reaven PD, Newell M, Rivas S, Zhou X, Norman GJ, Zhou JJ. Initiation of continuous glucose monitoring is linked to improved glycemic control and fewer clinical events in type 1 and type 2 diabetes in the veterans health administration. Diabetes Care. 2023;46(4):854‐863. 36807492 10.2337/dc22-2189 PMC10260873

[17] Moon SJ, Kim KS, Lee WJ, Lee MY, Vigersky R, Park CY. Efficacy of intermittent short-term use of a real-time continuous glucose monitoring system in non-insulin-treated patients with type 2 diabetes: a randomized controlled trial. Diabetes Obes Metab. 2023;25(1):110‐120. 36053813 10.1111/dom.14852

[18] Aleppo G, Beck RW, Bailey R, et al The effect of discontinuing continuous glucose monitoring in adults with type 2 diabetes treated with basal insulin. Diabetes Care. 2021;44(12):2729‐2737. 34588210 10.2337/dc21-1304 PMC8669539

[19] American Diabetes Association Professional Practice Committee . 7. diabetes technology: standards of care in diabetes—2025. Diabetes Care. 2025;48(Supplement_1):S146‐S166. 39651978 10.2337/dc25-S007 PMC11635043

[20] Yoo JH, Kim G, Lee HJ, Sim KH, Jin SM, Kim JH. Effect of structured individualized education on continuous glucose monitoring use in poorly controlled patients with type 1 diabetes: a randomized controlled trial. Diabetes Res Clin Pract. 2022;184:109209. 35065101 10.1016/j.diabres.2022.109209

[21] Healy SJ, Black D, Harris C, Lorenz A, Dungan KM. Inpatient diabetes education is associated with less frequent hospital readmission among patients with poor glycemic control. Diabetes Care. 2013;36(10):2960‐2967. 23835695 10.2337/dc13-0108 PMC3781555

[22] Dungan K, Lyons S, Manu K, et al An individualized inpatient diabetes education and hospital transition program for poorly controlled hospitalized patients with diabetes. Endocr Pract Off J Am Coll Endocrinol Am Assoc Clin Endocrinol. 2014;20(12):1265‐1273. 10.4158/EP14061.OR 25100371

[23] Magee MF, Baker KM, Bardsley JK, Wesley D, Smith KM. Diabetes to go-inpatient: pragmatic lessons learned from implementation of technology-enabled diabetes survival skills education within nursing unit workflow in an urban, tertiary care hospital. Jt Comm J Qual Patient Saf. 2021;47(2):107‐119. 33358126 10.1016/j.jcjq.2020.10.007

[24] Wexler DJ, Beauharnais CC, Regan S, Nathan DM, Cagliero E, Larkin ME. Impact of inpatient diabetes management, education, and improved discharge transition on glycemic control 12 months after discharge. Diabetes Res Clin Pract. 2012;98(2):249‐256. 23036785 10.1016/j.diabres.2012.09.016 PMC3514591

[25] Chang R, Piya MK, Ara P, Fernandes B, Simmons D. Use of glucose sensors for post-discharge care triaging of insulin-treated patients with type 2 diabetes: a feasibility study. Intern Med J. 2024;54(10):1739‐1743. 39230209 10.1111/imj.16494

[26] Depczynski B, Poynten A. Acceptance and effect of continuous glucose monitoring on discharge from hospital in patients with type 2 diabetes: open-label, prospective, controlled study. JMIR Diabetes. 2022;7(2):e35163. 35532995 10.2196/35163 PMC9127644

[27] Jaiswal R, Zhang M, Zuniga S, Myers AK. Integration of flash glucose monitoring during the transition of care from inpatient to outpatient settings in patients with type 2 diabetes. J Endocr Soc. 2021;5(Supplement_1):A427‐A428.

[28] Jenkins M, Simpson J, Ursuy T, Hanks J, Burroughs TE. Transitions of care from hospital to home: can continuous glucose monitoring improve outcomes for patients with diabetes? Sci Diabetes Self Manag Care. 2024;50(5):394‐405. 39297338 10.1177/26350106241268479

[29] Tian T, Aaron RE, Seley JJ, et al Use of continuous glucose monitors upon hospital discharge of people with diabetes: promise, barriers, and opportunity. J Diabetes Sci Technol. 2024;18(1):207‐214. 37784246 10.1177/19322968231200847 PMC10899827

[30] Zimet GD, Powell SS, Farley GK, Werkman S, Berkoff KA. Psychometric characteristics of the multidimensional scale of perceived social support. J Pers Assess. 1990;55(3-4):610‐617. 2280326 10.1080/00223891.1990.9674095

[31] Norman CD, Skinner HA. eHEALS: the eHealth literacy scale. J Med Internet Res. 2006;8(4):e27. 17213046 10.2196/jmir.8.4.e27 PMC1794004

[32] McNaughton CD, Cavanaugh KL, Kripalani S, Rothman RL, Wallston KA. Validation of a short, 3-item version of the subjective numeracy scale. Med Decis Mak Int J Soc Med Decis Mak. 2015;35(8):932‐936. 10.1177/0272989X15581800 PMC4592371 25878195

[33] Charlson ME, Pompei P, Ales KL, MacKenzie CR. A new method of classifying prognostic comorbidity in longitudinal studies: development and validation. J Chronic Dis. 1987;40(5):373‐383. 3558716 10.1016/0021-9681(87)90171-8

[34] Umpierrez GE, Castro-Revoredo I, Moazzami B, et al Randomized study comparing continuous glucose monitoring and capillary glucose testing in patients with type 2 diabetes after hospital discharge. Endocr Pract. 2025;31(3):286‐291. 39694328 10.1016/j.eprac.2024.11.018 PMC12233000

[35] O’Connor MJ, Ding X, Hernandez C, Hubacz L, Church RJ, O’Connor L. A pilot trial of continuous glucose monitoring upon emergency department discharge among people with diabetes Mellitus. Endocr Pract. 2024;30(2):122‐127. 37952581 10.1016/j.eprac.2023.11.001

[36] Clark TL, Polonsky WH, Soriano EC. The potential impact of continuous glucose monitoring use on diabetes-related attitudes and behaviors in adults with type 2 diabetes: a qualitative investigation of the patient experience. Diabetes Technol Ther. 2024;26(10):700‐708. 38526557 10.1089/dia.2023.0612

[37] Nassar CM, Montero A, Magee MF. Inpatient diabetes education in the real world: an overview of guidelines and delivery models. Curr Diab Rep. 2019;19(10):103. 31515653 10.1007/s11892-019-1222-6

[38] Demographics of Mobile Device Ownership and Adoption in the United States. Pew Research Center; 2024. Accessed 21 May 2025. https://www.pewresearch.org/internet/fact-sheet/mobile/

[39] Morris-Murray M, Frazzitta M. Using continuous glucose monitoring to measure and improve quality metrics: updates on the healthcare effectiveness data and information set 2024 glucose management indicator measure. J Manag Care Spec Pharm. 2024;30(10-b Suppl):S30‐S39. 39347972 10.18553/jmcp.2024.30.10-b.s30 PMC11443976

[40] Hall T, Warman MK, Oser T, et al Clinician-reported barriers and needs for implementation of continuous glucose monitoring. J Am Board Fam Med. 2024;37(4):671‐679. 39455273 10.3122/jabfm.2024.240049R1

[41] Rickards GM, Harrod JC, Del Valle K, Caballero AE, Palermo NE, McDonnell ME. Addressing inequity in continuous glucose monitoring access: leveraging the hospital in the Continuum of care. J Diabetes Sci Technol. 2024;18(6):19322968241288917. 10.1177/19322968241288917 PMC11574776 39558481

[42] Mulinacci G, Alonso GT, Snell-Bergeon JK, Shah VN. Glycemic outcomes with early initiation of continuous glucose monitoring system in recently diagnosed patients with type 1 diabetes. Diabetes Technol Ther. 2019;21(1):6‐10. 30575413 10.1089/dia.2018.0257

[43] Champakanath A, Akturk HK, Alonso GT, Snell-Bergeon JK, Shah VN. Continuous glucose monitoring initiation within first year of type 1 diabetes diagnosis is associated with improved glycemic outcomes: 7-year follow-up study. Diabetes Care. 2022;45(3):750‐753. 35018417 10.2337/dc21-2004

[44] Mann EA, Rompicherla S, Miyazaki B, et al Early continuous glucose monitor use in children and adolescents with type 1 diabetes: rates of initiation and impact on glycemic outcomes. Diabetes Care. 2025;48(5):768‐775. 40009551 10.2337/dc25-0076 PMC12034905

[45] Anderson TS, Yeh RW, Herzig SJ, et al Trends and disparities in ambulatory follow-up after cardiovascular hospitalizations: a retrospective cohort study. Ann Intern Med. 2024;177(9):1190‐1198. 39102715 10.7326/M23-3475 PMC11962735

[46] Anderson TS, Ayanian JZ, Herzig SJ, Souza J, Landon BE. Gaps in primary care follow-up after hospital discharge among medicare beneficiaries. J Am Geriatr Soc. 2025;73(7):2106‐2116. 40317736 10.1111/jgs.19496 PMC12303739

[47] Khodneva Y, Levitan EB, Arora P, Presley CA, Oparil S, Cherrington AL. Disparities in postdischarge ambulatory care follow-up among medicaid beneficiaries with diabetes, hospitalized for heart failure. J Am Heart Assoc. 2023;12(12):e029094. 37284763 10.1161/JAHA.122.029094 PMC10356027

